# Polarization Dependent Excitation and High Harmonic Generation from Intense Mid-IR Laser Pulses in ZnO

**DOI:** 10.3390/nano11010004

**Published:** 2020-12-22

**Authors:** Richard Hollinger, Paul Herrmann, Viacheslav Korolev, Maximilian Zapf, Valentina Shumakova, Robert Röder, Ingo Uschmann, Audrius Pugžlys, Andrius Baltuška, Michael Zürch, Carsten Ronning, Christian Spielmann, Daniil Kartashov

**Affiliations:** 1 Institute of Optics and Quantum Electronics, Friedrich-Schiller-University Jena, Max-Wien-Platz 1, 07743 Jena, Germany; p.herrmann@uni-jena.de (P.H.); viacheslav.korolev@uni-jena.de (V.K.); ingo.uschmann@uni-jena.de (I.U.); mwz@berkeley.edu (M.Z.); christian.spielmann@uni-jena.de (C.S.); daniil.kartashov@uni-jena.de (D.K.); 2 Helmholtz Institute Jena, Fröbelstieg 3, 07743 Jena, Germany; 3 Institute for Solid State Physics, Friedrich-Schiller-University Jena, Max-Wien-Platz 1, 07743 Jena, Germany; maximilian.zapf@uni-jena.de (M.Z.); robert.roeder@uni-jena.de (R.R.); carsten.ronning@uni-jena.de (C.R.); 4 Institute for Photonics, Technical University Vienna, Gußhausstrasse. 25-29, 1040 Vienna, Austria; valentina.shumakova@tuwien.ac.at (V.S.); audrius.pugzlys@tuwien.ac.at (A.P.); andrius.baltuska@tuwien.ac.at (A.B.); 5 Fritz Haber Institute, Faradayway 4-6, 14195 Berlin, Germany; 6 Department of Chemistry, University of California Berkeley, 237B Hildebrand Hall, Berkeley, CA 94720, USA; 7 Lawrence Berkeley National Laboratory, Materials Sciences Division, Berkeley, CA 94720, USA; 8 Abbe Center of Photonics, Friedrich Schiller University, Jena, Albert Einstein Straße 6, 07745 Jena, Germany

**Keywords:** high harmonic generation (HHG), ZnO, thin film, ellipticity dependence, tunneling excitation

## Abstract

The generation of high order harmonics from femtosecond mid-IR laser pulses in ZnO has shown great potential to reveal new insight into the ultrafast electron dynamics on a few femtosecond timescale. In this work we report on the experimental investigation of photoluminescence and high-order harmonic generation (HHG) in a ZnO single crystal and polycrystalline thin film irradiated with intense femtosecond mid-IR laser pulses. The ellipticity dependence of the HHG process is experimentally studied up to the 17th harmonic order for various driving laser wavelengths in the spectral range 3–4 µm. Interband Zener tunneling is found to exhibit a significant excitation efficiency drop for circularly polarized strong-field pump pulses. For higher harmonics with energies larger than the bandgap, the measured ellipticity dependence can be quantitatively described by numerical simulations based on the density matrix equations. The ellipticity dependence of the below and above ZnO band gap harmonics as a function of the laser wavelength provides an efficient method for distinguishing the dominant HHG mechanism for different harmonic orders.

## 1. Introduction

The nonlinear optical properties of zinc oxide (ZnO) crystals and nanostructures have been extensively studied over the last few decades. Light conversion processes such as second (SHG) and third harmonic generation (THG) as well as multiphoton absorption have been demonstrated in a wide variety of morphologies, such as micro- and nanowires, thin films, nanoribbons or nanoparticles [1,2,3,4]. A common feature of all these experiments is that the observed processes can be understood in the frame of perturbative nonlinear optics. In this regime, the laser field is considered as a weak perturbation and the induced nonlinear polarization can be described by a power series of the electric field [5]. However, for increasing laser field strength the power series fails to converge and the non-perturbative nonlinear optics regime is reached [6]. In this case, the electric field significantly perturbs the electronic structure of the material, enabling interband tunneling of an electron from the valence (VB) to the conduction band (CB) [7,8]. Furthermore, as a result of the strong electric field, coherent electron and hole dynamics are driven, which lead to intra- and interband high harmonic generation (HHG) via nonlinear intraband currents and polarization fields upon electron-hole recombination, respectively [9]. After the first experimental demonstration of HHG from ultrashort, mid-IR laser pulses in ZnO [10], the dependence of HHG on the ZnO lattice symmetry [11,12] and HHG from tailored ZnO nanostructures [13,14,15,16] have been investigated. Furthermore, HHG in ZnO was used to determine the carrier envelope phase (CEP) of few cycle mid-IR laser pulses [17].

The ellipticity of the electric field in the laser pulse is one of the key parameters determining the physics of HHG due to the nature of coherent electric field driven electron wave packet dynamics. In gases, control over the laser ellipticity provided a fundamental insight into the nature of the generation mechanism and the sharp drop in yield for elliptical fields enables isolated attosecond pulse generation using the polarization gating technique [18]. Experimental, as well as theoretical, investigations on ellipticity dependence of HHG in bulk semiconductors were carried out for materials having highly symmetric cubic lattices, such as crystalline Silicon, zinc blend ZnS or cubic MgO [19,20,21]. However, in wurtzite lattice materials, like ZnO, the ellipticity dependence were only investigated theoretically so far [22,23].

In this work, we present an experimental investigation of the ellipticity dependent photoluminescence (PL) and HHG in the wurtzite lattice and large band gap semiconducting ZnO. Both processes, PL and HHG, are initiated by tunneling excitation in the strong electric field of a mid-IR laser pulse and both are directly related to the carrier density in the CB. Thus, it allows us to use the ellipticity dependence of the PL for monitoring the tunneling excitation efficiency. We use polycrystalline thin film and bulk c-cut ZnO crystal samples to distinguish between the effects of propagation and reabsorption of the high harmonic signal in the material. We demonstrate that the intraband and interband mechanisms of generation have qualitatively different sensitivity to the ellipticity of the laser polarization, identifying the dominant generation mechanism for individual harmonics.

## 2. Materials and Methods

Polycrystalline ZnO thin films were synthesized by RF-magnetron sputtering on sapphire substrates. Scanning electron microscope (SEM) and X-ray diffraction (XRD) characterization of the samples reveal a 300 nm thick film with columnar crystalline grains, grown in the <001> direction, with a size of 120–150 nm, as published in [24]. The polished ZnO single crystal sample exhibits a thickness of 100 µm and a crystalline orientation of <001>. The ZnO samples were irradiated with 100 fs long mid-IR laser pulses in the spectral range from 3–4 µm, which were generated by non-collinear difference frequency generation (NDFG) between the signal and idler pulses of an optical parametric amplifier (OPA). The OPA was pumped by 5 mJ, 35 fs laser pulses with a central wavelength at 800 nm and a pulse repetition rate of 1 kHz. A pair of wire grid polarizers and a broadband *λ*/4-plate were used to attenuate the laser power and adjust the polarization ellipticity, respectively. The laser beam was focused by a *f* = 10 cm CaF_2_ lens onto the samples oriented such that the laser propagation direction was along the c-axis. The generated signals were collected in a transmission geometry and refocused onto the entrance slit of a spectrometer using a *f* = 5 cm CaF_2_ lens. The mid-IR pulse duration and laser spot size in the plane of the ZnO sample were determined using a frequency resolved optical gating (FROG) [25] and a knife-edge technique, respectively. The laser intensity was then estimated from the experimentally determined pulse energy, duration and spot size. All experiments were carried out at room temperature and in an ambient atmosphere.

## 3. PL and HHG Emission from ZnO Thin Films and Bulk

**Figure 1:**
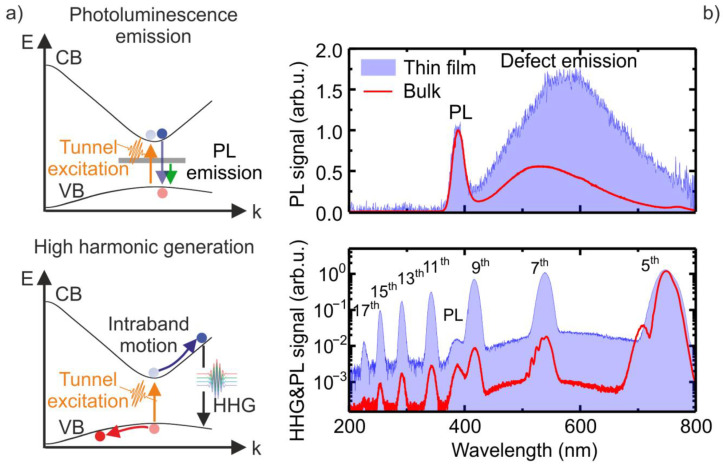
Photoluminescence (PL), defect emission and high harmonic generation (HHG) in ZnO. (**a**) Schematic illustration of recombination processes upon strong field tunneling excitation of an electron-hole pair. Upper panel: PL and defect emission as a result of electron-hole recombination from conduction band (purple arrow) and defect states (green arrow). Lower panel: Harmonic radiation is generated by a field driven nonlinear intraband motion of the electron as well as electron-hole recombination. (**b**) Emission from a thin film and bulk sample irradiated with linear polarized femtosecond laser pulses with a central wavelength at 3.85 µm. Upper panel: Normalized photoluminescence and defect emission spectra from the thin film (blue) and bulk (red) sample. Lower panel: High harmonic spectrum, on top of the PL and defect emission, generated in a thin film (blue) and bulk sample (red).

Irradiating ZnO with intense mid-IR light in the spectral range from 3–4 µm corresponds to a band gap (3.2 eV) to photon energy (0.4–0.3 eV) ratio in the range of 8 to 10. According to the theory of strong field excitation in solids, the regime of excitation is determined by the Keldysh parameter $\gamma =\frac{2\pi \;c}{\lambda \;e\;E_{L}}\sqrt{m^{*}E_{g}}$ [26], with multiphoton absorption or tunneling being the dominating excitation mechanism for γ>1 or γ<1, respectively. Here, c is the speed of light, e the electron charge, $m^{*}$ is the electron effective mass, $E_{g}$ is the bandgap, $E_{L}$ is the laser pulse electric field strength and *λ* is the laser wavelength. We estimate a Keldysh parameter of γ < 0.8 for the above-mentioned experimental parameters, thus tunneling from the VB to the CB is the more dominant excitation mechanism. Excited electrons eventually recombine via direct band gap recombination or involving deep defect levels caused near band edge (NBE) PL and defect emission (Figure 1a, upper panel). Nonlinear periodic intraband currents can be driven by strong laser field motion of electrons and holes in the CB and the VB, respectively, resulting in the generation of higher order harmonics of the fundamental laser frequency. Furthermore, higher harmonics can be generated via interband recombination of electrons and holes, leading to emission of quanta with energies determined by the electron-hole momentum at the moment of recombination plus the bandgap energy (Figure 1a, lower panel) [23]. Thus, this mechanism would be responsible for harmonics with energies of quanta larger than the bandgap.

Figure 1b shows the PL and defect emission (upper panel) as well as the HHG spectra (lower panel) from the ZnO thin film (blue) and bulk (red) sample irradiated with linear polarized laser pulses at 3.85 µm wavelength. All spectra shown in Figure 1b were measured under identical conditions. For the NBE PL and defect emission measurements, shown in the upper panel, the harmonic radiation, propagating along the driving mid-IR laser beam, was blocked by a small beam block placed in the beam center after the collecting optics, whereas the PL is isotropically emitted. Both samples show two dominant emission bands at 385 nm and around 550 nm (see upper panel of Figure 1b), which originate from NBE PL and defect state-related recombination [27]. The thin film sample shows a three times larger defect emission to PL signal ratio compared to the bulk sample. Harmonics from the 5th up to the 17th order were detected from both samples. However, the intensities of higher harmonics above the 5th order, normalized to the 5th harmonic intensity, are much weaker in the bulk sample compared to the thin film. On the other hand, the absolute intensity of the 5th harmonic from the bulk was about 500 times higher compared to the thin film sample. Furthermore, the 5th and 7th harmonics generated in the bulk sample show a clear modulation which is absent in the spectra obtained from the film sample. These modulations and the relative drop of the harmonic intensities in the bulk sample can be explained by propagation effects. The modulations seem to originate from the harmonic radiation generated at the front sample-air interfaces, which is interfering with reflections at the rear end [17]. Since the absorption length of ZnO at 360 nm and 560 nm is 0.3 and 3 µm, respectively [28], the strong relative drop of the harmonic intensities for orders larger than the 5th is addressed to reabsorption of the harmonic signal by interband and defect state transitions.

## 4. Ellipticity Dependence of Strong Field Light Absorption

**Figure 2:**
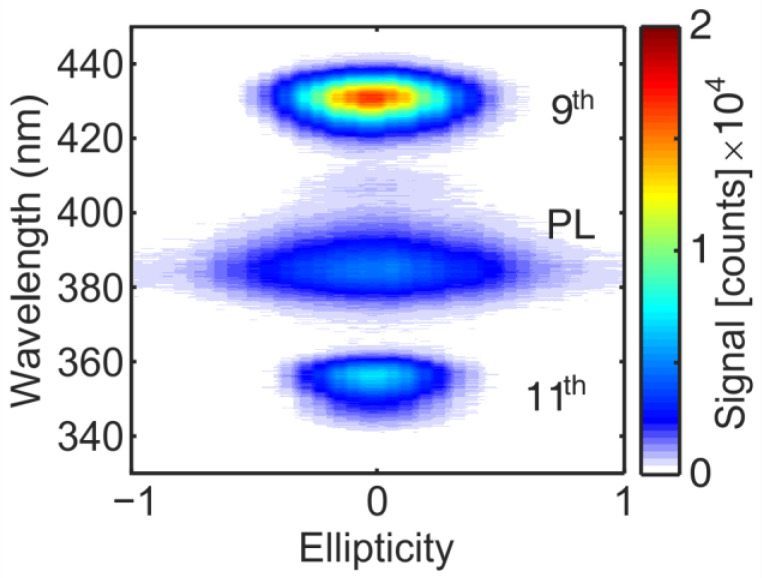
Photoluminescence (PL) and high harmonic emission from a ZnO thin film irradiated with femtosecond laser pulses at 3.8 µm as a function of the laser ellipticity.

Figure 2 shows the spectra of PL, 9th and 11th harmonic emission from a ZnO thin film irradiated at 3.8 µm as a function of the laser ellipticity. The PL and HHG yields are highest when the laser field is linearly polarized (*ε* = 0) and drop when the laser polarization is changed from elliptical (0 < |*ε*| <1) to circular polarization (|*ε*| = 1). While the HHG signal vanishes completely for a certain laser ellipticity, the PL signal drops by a ratio of 5.8 when the laser polarization is changed from linear to circular. The PL signal depends on the number of excited electrons and thus can monitor the laser polarization-dependent efficiency of light absorption at the excitation wavelength. The use of thin films to study the polarization dependence of light absorption is important to rule out nonlinear propagation effects like self-focusing, which can be present in the bulk sample [29]. A previous work [24], using the same thin films, showed the dependence of three photon absorptions of near infrared light (0.8 µm) on the laser ellipticity. There, the PL signal decreases by a factor 1.8 when circular compared to when linear polarized laser light was used. In the present case, the reduction of the emission signal, i.e., light absorption, is much stronger for long wavelength light when carrier excitation is caused by tunnel excitation. In the case of nonlinear light absorption via multiphoton processes, the drop in the absorption efficiency was explained by laser polarization and depended on interband transition rules to higher laying bands [30]. The coupling to higher laying CB plays a minor role due to the small photon energy in the case of long wavelength light absorption. However, the tunneling rate $w_{Tun}\sim \exp\left[\;-∆E^{3/2}/E_{L}\;\right]$ depends exponentially on the electric field strength of the laser $E_{L}$ and the energy difference ∆E between the ground and excited state. When the laser polarization is changed from linear to circular, the electric field strength reduces by a factor of $\sqrt{2}$. Thus, one might expect that the exponential dependence of the tunneling rate on the electric field strength may lead to the drop of the PL intensity. However, this exponential dependence is softened because of the following reason: in the so-called quasi-static limit, the electric field strength in the expression for the tunneling rate can be replaced by the time-dependent electric field amplitude in the laser electromagnetic wave [31]. Therefore, the excitation occurs only in the vicinity of maxima in the modulus of the field amplitude for the situation of time alternating linearly in polarized fields. In the case of circular polarization, the electric field modulus is constant in time and rotates in space with the laser frequency. Thus, tunnel excitation in a circular polarized field proceeds like in a static field, during the whole duration of the optical cycle.

## 5. Ellipticity Dependence of HHG

**Figure 3:**
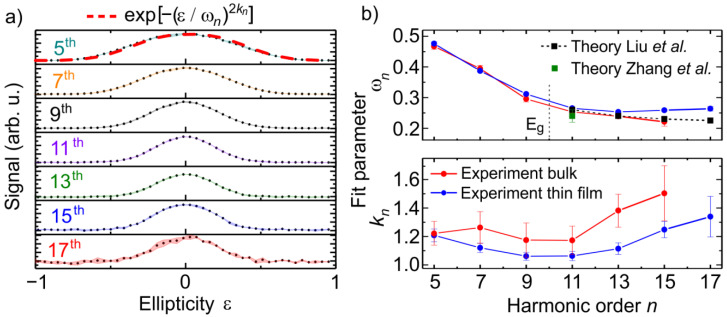
Dependence of HHG in ZnO on the laser ellipticity for a laser intensity of 0.39 TW/cm^2^. (**a**) Spectrally integrated emission of the 5th up to the 17th harmonic from the ZnO thin film sample. (**b**) Upper panel: Comparison of the experimentally determined sensitivity of the detected harmonic orders *n* on the laser ellipticity, described by $\omega_{n}$, with theoretically determined values from Liu et al. [22] and Zhang et al. [23]. Lower panel: Fit parameter $k_{n}$ as a function of the detected harmonic order n from the bulk (red) thin film sample (blue).

Comparing the ellipticity dependence of the high harmonic and PL emission, we conclude that the laser ellipticity affects the HHG process not only via a reduced electron excitation rate (see Figure 2). Additionally, it seems that different harmonic orders (9th and 11th harmonics, for example) show also different sensitivities on the laser ellipticity. The spectrally integrated yields of the 5th up to the 17th harmonics, generated in ZnO thin films by laser pulses with a wavelength of 3.85 µm and an intensity of 0.39 TW/cm^2^, are shown in Figure 3a as a function of the laser ellipticity. All harmonic orders are most efficiently generated for linearly polarized laser pulses and vanish for circularly polarization. However, the drop of the harmonic intensities with increasing ellipticity depends on the harmonic order. In order to describe the dependence of the HHG process on the laser ellipticity, a super-Gaussian fit-function $\exp\left[-{\left(\varepsilon /\omega_{n}\right)}^{2k_{n}}\right]$ was used, in accordance with the theory work published in reference [22]. The waist, determined by the fit parameter $\omega_{n}$, is used for a quantitative characterization of the sensitivity of the HHG process on the laser ellipticity for different harmonic orders *n*. Figure 3b shows the fit parameters $\omega_{n}$ and $k_{n}$, determined from the fitting procedure of the experimental results measured in the ZnO bulk (red) and thin film (blue) samples as a function of the harmonic order *n*. Furthermore, fit parameter values—calculated using theoretical models by Liu et al. (black) [22] and Zhang et al. (green) [23]—using a Gaussian fit function $\left(k_{n}=1\right)$ are shown in the upper panel of Figure 3b. Both theoretical simulations are based on the density matrix equation and the results for the above band gap higher harmonics are interpreted in a frame of the semiclassical saddle-point analysis. The vertical dashed line depicts the ZnO bandgap at 3.2 eV. As it follows from the Figure 3b (upper panel), the ellipticity dependence of $\omega_{n}$ for below and above bandgap higher harmonics varies from 0.48 to 0.23 for the 5th and the 15th harmonic, respectively. The below band gap harmonics are much less sensitive to the laser ellipticity, corresponding to a larger $\omega_{n}$, than the above band gap harmonics. Qualitatively similar observations have been previously made experimentally [10] as well as theoretically [22]. Best fitting was achieved for a parameter $k_{n}$ > 1 value for all harmonics (see Figure 3b, lower panel). In combination with a larger coefficient of determination (*R*^2^), this motivated the use of the super-Gaussian fit function. Please note that the Gaussian curve waist is invariant under changes of the parameter $k_{n}.$ Therefore the additional fit parameter $k_{n}\;$has no influence on the comparison with the work of Liu et al. (as shown in the upper panel of Figure 3b), in which $k_{n}$ = 1 was used. Comparison of the sensitivity of the HHG-process in the bulk (red) and the thin film samples (blue) on the laser ellipticity reveal an identical behavior for harmonics below the 9th order. However, for higher harmonic orders the tendency varies: The HHG process in the bulk sample becomes more sensitive to the laser ellipticity with increasing harmonic order. In contrast, in the film sample, the generation of the 13th order is most sensitive on the laser ellipticity, compared to the lower and higher orders. The experimental results measured for the bulk sample are in very good agreement with numerical simulations published in reference [22]. The polycrystalline structure, a different impurity level and an associated modification of the electronic structure may explain the small deviations between the theory and experimental values obtained from the thin film. The significant effect of the electronic structure on the ellipticity dependence of HHG in ZnO was demonstrated by Liu et al. [22] In that work it is shown that small modifications of the material band structure qualitatively allows us to reproduce the measured ellipticity dependence of HHG in the thin film sample.

**Figure 4:**
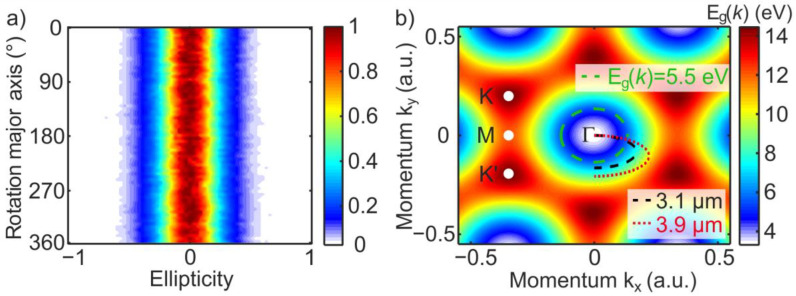
Effect of the electronic structure symmetry properties on the ellipticity dependence of the HHG process. (**a**) Ellipticity dependence of the 11th harmonic as a function of the laser polarization major axis rotation around the ZnO crystal c-axis. (**b**) Electron momentum-dependent band gap. The high symmetry points Γ, K, K’ and M are labelled. The area from the Γ-point to the green dashed circle highlights the condition $E_{g}$($\vec{k}$) < 5.5 eV. This is the region where experimentally detectable harmonics are generated. The black dashed and red dotted lines indicate the electron trajectory upon strong field interaction when a 3.1 and 3.9 µm laser and an ellipticity of 0.5 is used, respectively.

In references [19,20,21] it was shown that the ellipticity dependence of HHG is sensitive to the lattice symmetry. We have investigated the ellipticity dependence of the HHG process in the ZnO single crystal by rotating the laser polarization major axis in respect to the c-axis. Figure 4a shows the surprising result of the same ellipticity dependence of the 11th harmonic while rotating the polarization major axis of the laser pulses. Considering the band gap $E_{g}$($\vec{k}$) of ZnO, as shown in Figure 4b, a periodic oscillation due to the 6-fold symmetry of the hexagonal electronic structure is expected. The electron quasi momentum $\vec{k}$ dependent band gap $E_{g}$($\vec{k}$) = $E_{CB}$($\vec{k}$) − $E_{VB}$($\vec{k}$) (as shown in Figure 4b) is defined by the CB and VB energies, according to the expressions and parameters given in reference [23]. To explain our observation, the highest detected harmonic order (17th) with the energy of quanta 5.5 eV is considered. As it was pointed out before, the above band gap harmonics originate from an electron-hole recombination at a position in the reciprocal space far from the Γ-point [9,32]. As illustrated by the green dashed circle in Figure 4b, the recombination of electrons responsible for emission of quanta with energies in the range 3.2–5.5 eV corresponds to the generation of the above bandgap harmonics up to the 17th order and appears at positions where the electronic structure is almost isotropic, i.e., electron and hole trajectories are invariant in respect to the rotation of the laser polarization major axis. To observe the symmetry-induced modulations in ellipticity dependence, measurements of higher harmonics above the 21st order are necessary [23]. These harmonics belong to the VUV spectral range and could not be measured due to experimental constrains. Please note, in this work only c-plane oriented ZnO is investigated. Using a different lattice plane orientation will lead to a more complex ellipticity dependence due to the material birefringence and the contribution of electric field components parallel and perpendicular to the material c-axis [11,12].

**Figure 5:**
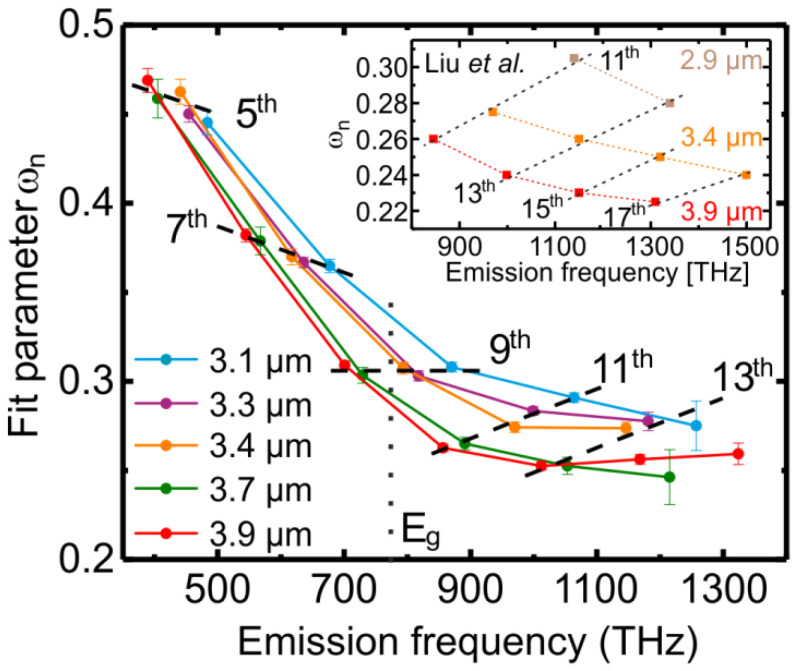
Sensitivity of HHG from a ZnO thin film on the laser ellipticity as a function of the harmonic order and the emission frequency for five laser wavelengths. The laser intensity in the experiment was fixed to 0.39 TW/cm^2^. The inset depicts simulations performed by Liu et al. [22].

Finally, we investigated how the ellipticity dependence changes with the wavelength of the excitation laser. Figure 5 shows the fit parameter $\omega_{n}$ as a function of the harmonic orders and corresponding harmonic frequencies for five different laser wavelengths between 3.1 and 3.9 µm. Here, only the thin film results are shown to avoid additional complications caused by reabsorption and interference effects in the bulk sample (see Figure 1b). The decreasing slope of the fit parameter for the below band gap 5th and 7th harmonics (dashed lines) indicates their increasing sensitivity when shortening the laser wavelength from 3.9 to 3.1 µm. The width of the ellipticity dependence of the near bandgap 9th harmonic is almost independent on the laser wavelength. At last, the above band gap harmonics (11th and 13th) are less sensitive with respect to the ellipticity when a shorter laser wavelength is used for HHG. Furthermore, comparing the values of the fit parameter for an emission frequency of 850 THz (Figure 5), corresponding to the 9th harmonic of 3.1 µm (bright blue) and 11th harmonic of 3.9 µm (red), we conclude that it is not the harmonic frequency but the laser wavelength that defines the sensitivity of the HHG process on the laser ellipticity. Thus, the physics of the generation process rather than the energy of the emitted quanta of the harmonics ($E_{n}$) matters for the ellipticity dependence of HHG. The inset in Figure 5 shows simulation results based on the model suggested in reference [22] for three different driving wavelengths, demonstrating good quantitative agreement with the results of our experimental measurements. Since the above band gap higher harmonics ($E_{n}$ > $E_{g}$) are generated by interband transitions, the strong ellipticity dependence on the laser wavelength must rely on the recollisional nature of the HHG process. As was shown by Zhang et al. [23], the laser ellipticity strongly modifies the electron (hole) trajectory and thus the electron-hole recombination time. Furthermore, using a semiclassical description of the recollision model, Vampa et al. [33] demonstrated the strong wavelength dependence of the interband HHG process.

According to simulations, the below band gap higher harmonics are dominantly generated by intraband transitions [9,22]. The intraband higher harmonic spectrum is defined by the Fourier transformation of the temporal derivative of the intraband current that can be calculated as $\frac{d\vec{j}}{dt}=\frac{e}{ћ}∆E_{g}{}^{-1}|{\;}_{\vec{k}=\vec{k}\left(t\right)}{\vec{E}}_{L}\left(t\right)$ [20], given by the product from the band structure curvature and the laser field strength ${\vec{E}}_{L}\left(t\right)$. Figure 4b shows the electron trajectory within half an optical cycle in the reciprocal space generated by 3.1 (black dashed line) and 3.9 µm laser light fields (red dotted lines) with an intensity of 0.35 TW/cm^2^ corresponding to 0.15 V/Å field strength and a laser ellipticity of 0.5. Here, the Bloch acceleration theorem $\vec{k}\left(t\right)=\frac{e}{ћ}\int_{t_{0}}^{t}{\vec{E}}_{L}\left(t^{\prime }\right)dt^{\prime }$ was used to determine the electron quasi momentum. Furthermore, it was assumed that the electron is generated at the Γ-point at a time $t_{0}$, when the electric field reaches its maximum value. Higher harmonics are only generated when the curvature of the electronic structure is not constant, i.e., when the band is non-parabolic. The band structure curvature of ZnO varies stronger for momentum values $\left|\vec{k}\right|$ with a large distance to the Γ-point. As shown in Figure 4b, an elliptically polarized laser field leads to a turn of the electron trajectory. Larger k-values, which are linked to an inhomogeneous electron movement, are thus less likely reached. Furthermore, for higher laser ellipticity an increasing part of the electron trajectory undergoes a homogeneous movement during a half cycle of the laser field, i.e., $∆E_{g}{}^{-1}|{\;}_{\vec{k}=\vec{k}\left(t\right)}=constant,$ resulting in a less efficient HHG. This effect is more pronounced for short wavelength lasers, since the laser field-based momentum transfer (quiver momentum) is directly proportional to the laser wavelength. Thus, the intraband generation mechanism is strongly affected by the laser ellipticity for short driving wavelengths, which is in good agreement with our findings, as displayed in Figure 5.

## 6. Conclusions

We report on the experimental investigations of the ellipticity dependence of the NBE PL and HHG in II-IV semiconductors with wurtzite lattice symmetry, exemplarily using ZnO thin film and bulk samples irradiated with intense mid-IR femtosecond laser pulses. We show that in the tunneling regime of strong field excitation in semiconductors the difference in the excitation rate between the linearly and circularly polarized fields in the laser pulse is significantly larger than in the case of multiphoton excitation. This enhanced difference might be explained by the exponential dependence of the transition probability on the electric field amplitude in the tunneling regime. The harmonic emission spectra generated in the bulk sample showed features resulting from the propagation of the harmonic radiation in the material. Thus, the spectra show a strong damping of higher harmonic radiation due to reabsorption. The measured ellipticity dependence of HHG in bulk ZnO appears to be an experimental verification of recently published numerical simulations [22,23]. A similar dependence is also observed in the thin film sample with minor deviations indicative for a modified electronic structure induced by the polycrystalline structure and defects of the film. Measurements with varying wavelengths of the driving mid-IR laser pulses suggest that it is the physical mechanism of HHG rather than the absolute harmonic emission wavelength, which determines the sensitivity of HHG on the laser ellipticity. In agreement with numerical simulations, based on the recently published theoretical models [22,23], the above band gap harmonics, generated by the interband mechanism, are less sensitive to the laser ellipticity for short wavelength drivers. In contrast, the below band gap harmonics, which are attributed to the intraband generation mechanism, show a stronger dependence on the laser ellipticity for shorter driving wavelengths.
